# Total Antioxidant Capacity; A Potential Biomarker for Non-Invasive Sex Prediction in Culture Medium of Preimplantation Human Embryos

**DOI:** 10.22074/cellj.2019.6115

**Published:** 2019-06-15

**Authors:** Nahid Nasiri, Leila Karimian, Fatemeh Hassani, Hamid Gourabi, Hiva Alipour, Zahra Zolfaghari, Poopak Eftekhari-Yazdi

**Affiliations:** 1 Department of Embryology, Reproductive Biomedicine Research Center, Royan Institute for Reproductive Biomedicine, ACECR, Tehran, Iran; 2 Department of Genetics, Reproductive Biomedicine Research Center, Royan Institute for Reproductive Biomedicine, ACECR, Tehran, Iran; 3 Biomedicine Group, Department of Health Science and Technology, Faculty of Medicine, Aalborg University, Aalborg, Denmark; 4 Department of Epidemiology and Reproductive Health, Reproductive Epidemiology Research Center, Royan Institute for Reproductive Biomedicine, ACECR, Tehran, Iran

**Keywords:** Antioxidant, Culture Medium, Glucose, Human Embryo, Sexuality

## Abstract

**Objective:**

The presence of a sex related metabolic difference in glucose utilization and, on the other hand, different developmental kinetic rates in human preimplantation embryos, has been previously observed, hawever, the correlation between these two events is unknown. Oxidative stress (OS) induced by higher glucose consumption appears to be a possible cause for the delayed development rate in female embryos. We examined the correlation between glucose consumption and total antioxidant capacity (TAC) concentration in individual embryo culture media for both male and female embryos.

**Materials and Methods:**

In this cross-sectional study, we evaluated high quality embryos from 51 patients that underwent intracytoplasmic sperm injection (ICSI) and preimplantation genetic diagnosis (PGD) at the Royan Institute between December 2014 and September 2017. The embryos were individually cultured in G-2TMmedium droplets at days 3-5 or 48 hours post PGD. We analysed the spent culture media following embryo transfer for total antioxidant capacity (TAC) and any remaining glucose concentrations through fluorometric measurement by chemiluminecence system which indirectly was used for measurement of glucose consumed by embryos.

**Results:**

The results showed that female embryos consumed more glucose which was associated with decreased TAC concentration in their culture medium compared to male embryos. The mean of glucose concentration consumed by the female embryos (30.7 ± 4.7 pmol/embryo/hour) was significantly higher than that of the male embryos (25.3 ± 3.3 pmol/embryo/hour) (P<0.001). There were significantly lower levels of TAC in the surrounding culture medium of female embryos (22.60 ± 0.19 nmol/µl) compared with male embryos (24.74 ± 0.27 nmol/µl, P<0.01).

**Conclusion:**

This finding highlighted the utilization of sex dependent metabolic diversity between preimplantation embryos for non-invasive sex diagnosis and suggests the TAC concentration as a potential noninvasive biomarker for prediction of sex.

## Introduction

Throughout the past few decades, preimplantation embryo physiology and its related technologies (proteomics and metabolomics) have been employed with multiple purposes. Better recognition of embryo properties, improvement of embryo culture media, and selection of the most viable embryos for transfer via in vitro fertilization/intracytoplasmic sperm injection (IVF/ICSI) cycles are the most important goals (1, 2). Assessment of embryo metabolism has been suggested recently for diagnosis of sex related differences between preimplantation embryos. These differences are attributed to different X chromosome content among males and females during the finite period-between embryonic genome activation and excess X chromosome inactivation (3). During this period, the presence of two transcriptionally active X chromosomes in females forms the basis for the different proteome and physiologies between males and females (4). These differences can lead to sex dimorphism including different concentrations of X-linked enzymes that are primarily involved in nutrient utilization and energy metabolism (5). There are numerous reports about the feasibility for assessing these metabolic differences in embryonic culture media without the need for embryo manipulation and increased time expenditure (4, 5) and in the future this rapid, non-invasive approach, may be able to replace preimplantation genetic diagnosis (PGD) which involves the biopsy of a blastomere at the cleavage/blastocyst stage followed by the identification of the sex chromosomes. PGD is considered an invasive, time consuming technique for sex identification in preimplantation human embryos prior to their transfer to the uterus (4). In order to quantify such physiological differences, we can analyze the X chromosome dependent events in embryo blastomeres. Based on possible data analysis, it may be feasible to predict an embryo’s sex without the use of PGD. Among the various metabolites, more attention has been paid to glucose which presents at high concentrations in the female reproductive tract during early embryo development. It has been suggested that glucose has a greater relationship with embryo sex compared to other metabolites (6). Previous reports discussed the different schema of glucose utilization between male and female preimplantation embryos (4). Initial studies reported increased glucose and pyruvate uptake by male embryos compared to females (7), whereas more recent studies reported increased glucose uptake by female embryos (5, 6).

The rate of glucose metabolism may change due to X-choromosome dosage mainly because the Glucose -6- phosphate dehydrogenase (G6PD), that catalyzes the principal glucose metabolism pathway (pentose phosphate pathway, PPP), is encoded by X-chromosome, and this double concentration in female blastocysts compared to male blastocysts (8). On the other hand, a slower development rate of female preimplantation embryos in the in vitro culture (IVC) has been frequently observed (9, 10). According to these studies, delays in development have shown significant correlation with increased glucose consumption (6). Further glucose consumption and hyperglycemia are commonly associated with reduced or delayed blastocyst formation (11), lower implantation rate (12), reduced live birth rate, and decreased fertility due to induction of metabolic disorders (13).

Several mechanisms proposed for such disorders attributed to high glucose consumption include increased cell apoptosis, glucose transport perturbation, and mitochondrial dysfunction (6), all of which may induce oxidative stress (OS) followed by increased reactive oxygen species (ROS) production (14). However, although female embryos experience a slower rate of development along with increased glucose consumption compared to male embryos, the correlation of the sex related glucose consumption with induced OS in culture medium that surrounds the embryo is unknown. Therefore, the present study is the first to investigate the relationship between glucose uptake on days 4 and 5 by individually cultured human embryos and the total antioxidant capacity (TAC) concentrations in their culture mediums and applying it to predict embryo sex.

## Materials and Methods

### Participants

This cross-sectional study included 60 cleavage-stage embryos from 51 fertile couples at the Royan Institute, Tehran, Iran, between December 2014 and September 2017. All 51 couples signed a written informed consent for the collection of residue embryo culture media after embryo transfer. For each couple prior to starting the treatment, a comprehensive counseling was provided by a reproductive endocrinologist and clinical geneticist. Thirty eight embryos from 30 women referred to Royan Institute for ICSI-PGD as an indication for the risk of sex linked diseases, and 22 embryos from 13 patients that underwent ICSI-PGD because of sex selection decision for family balancing (i.e., for patients who already had at least two children of one sex and desired a child of the other sex).

We performed ICSI in order to achieve high fertilization rates in included patients and prevent the formation of sperm bound to the zona pellucida during the blastomere biopsy. The local Ethics Committee of Royan Institute granted approval for this study (reference number: EC/91/1033). All data were collected following patient informed consent and protection of patient confidentiality. Throughout the duration of this study, all gamete and embryo culture media and handling protocols, as well as embryology lab staff remained constant.

### Ovarian hyperstimulation

Patients included in this study underwent standard controlled ovarian stimulation that consisted of suppression of pituitary gonadotropin secretion by subcutaneous injection (500 mg/d) of the gonadotropin releasing hormone (GnRH) agonist, buserelin acetate (Suprefact, Hoechst AG, Germany). Patients received these injections during the mid-luteal phase of the preceding ovarian cycle (day 21). We conducted this study from August 2014 to September 2015 at the Royan Institute’s Assisted Conception Unit. Once ovarian suppression was confirmed, ovarian stimulation was initiated with recombinant follicle stimulating hormone (FSH, Gonal F, SC injection, 150 IU/d, Serono, Switzerland). When the average diameter of at least three follicles reached 18 mm, each patient received a single injection of human chorionic gonadotropin (hCG) (10000 IU, Pregnyl, Organon, Netherlands). Oocyte collection was performed by standard ultrasound guided follicular puncture at 36 hours after the hCG trigger.

### Intracytoplasmic sperm injection and embryo culture

At 1 hour after oocyte retrieval, we selected morphologically ideal oocytes for ICSI. Oocytes were maintained in G-IVFTM medium (Vitrolife, Sweden) for approximately 2 hours before ICSI. The spermatozoa were prepared using density gradient centrifugation (AllGrad®, LifeGlobal, US). For ICSI, the oocytes were initially incubated in 80 IU/ml hyaluronidase for less than 30 seconds and cumulus cells were stripped off the oocyte by gentle pipetting. Fertilization was confirmed at 16 to 17 hours after ICSI, by the presence of two pronuclei and a second polar body. Zygotes were placed individually in 20 μl fresh G-1TM medium (Vitrolife) supplemented with 10% recombinant human serum albumin (HSAsolutionTM, Vitrolife) under oil (OVOILTM, Vitrolife) for a 48 hours culture.

### Embryo biopsy and preimplantation genetic diagnosis

Embryo biopsy was performed on day 3 after fertilization. Embryos of Grade A, B or C, that had >6 cells and <20% fragmentation were biopsied. For each selected embryo the blastomeres were checked for the presence of nuclei. Each embryo was placed in a droplet of Ca^2+^- and Mg^2+^-free medium (G-PGDTM, Vitrolife) and the zona pellucida was perforated using a Nikon TE300 inverted microscope (Nikon, Japan) equipped with a zona infrared laser optical system (ZILOS, Hamilton- Thorn, Beverg, MA) with a 1.48-mm infrared diode laser beam. One blastomere was gently aspirated with an aspiration pipette (± 35 μm outer diameter) and individually fixed under an inverted microscope. Sex chromosomes were assessed as previously described (15). We used DNA probes for chromosomes X and Y (Vysis, Abottmol, USA) for PGD analysis of the cells. The probe for the X chromosome was labeled with spectrum aqua and for the Y chromosome the probe was labeled with spectrum green which resulted in blue and green fluorescence, respectively. After the biopsy on day 3, embryos were individually cultured in 20 μl of G-2TM medium (Vitrolife) supplemented with 10% HSA until sex determination and transfer to uterus on day 5 (120 hours after fertilization).

### Measurement of glucose and total antioxidant capacity concentrations

To evaluation of sex related differences in glucose and TAC concentrations between male and females embryos, remaining 20 μl embryo culture media (10 μl for each variable) from all 48 hours cultured embryos (days 3 to 5 between embryo biopsy and embryo transfer) were used after transfer of embryos.

At the time of culture media evaluation, the embryo sexuality was unknown because the researcher was not informed from the sex determination results specified by PGD. Concentration analysis was based on fluorometric measurement of any remaining glucose using a chemiluminecence system (SynergyTM H4 Hybrid Multi- Mode Microplate Reader, Biotek, USA, Ex/Em=535/587 nm) and a glucose assay kit (K618-100, Biovision) which can detect 10 pmol to 10 nmol glucose per assay.

TAC concentration was evaluated via colorimetric measurement giving a broad absorbance peak around 570 nm and a TAC assay Kit (K274-100, Biovision, USA) which its detection limit is approximately 0.1 nmol per well (or 1 μM) of Trolox or TAC. Since the direct evaluation of glucose consumption by embryo is possible only through invasive techniques such as radioimmunoassay, in a non-invasive approach, we measured the amount of glucose consumed by the embryos through considering the glucose concentration that remained in culture medium after the 48 h culture period as well as concentration of glucose in the medium at the start of the incubation period (control), volume of individual embryo surrounded culture medium (20 μl) and the number of hours of embryo incubation (48 hours). In this way, for control and each embryo sample, the volume of culture medium multiplied by measured glucose concentration and the difference between the two time points (i.e. at the start of embryo incubation and 48 hours after incubation) was the number of pmols consumed by the embryo during the incubation. Then we divided this by the number of hours of incubation and final values were obtained in pmol/embryo/hours. accordingly our proposed formula for non-invasive measurement of glucose uptake by each embryo is as fallows;

[G]e=(([G]48h×V)-([G]0×V))h

Glucose concentration consumed per embryo (pmol/ embryo/hour): [G]_e_

Volume of culture medium (microliters): V

Glucose concentration at incubation time zero: [G]_0_

Glucose concentration at the end of incubation time (48 hours): [G]_48h_

 Incubation time duration (hours): h

### Statistical analysis

Comparison of quantitative variables (TAC and glucose concentrations) between the male and female groups was performed by the student’s t test for independent samples in normally distributed data, as assessed by the Kolmogorov-Smirnov test. P<0.01 was considered statistically significant. All data were expressed as mean ± standard error (SE). The statistical analysis was carried out using SPSS version 16 (SPSS Inc., Chicago, IL, USA).

## Results

**Fig.1:**
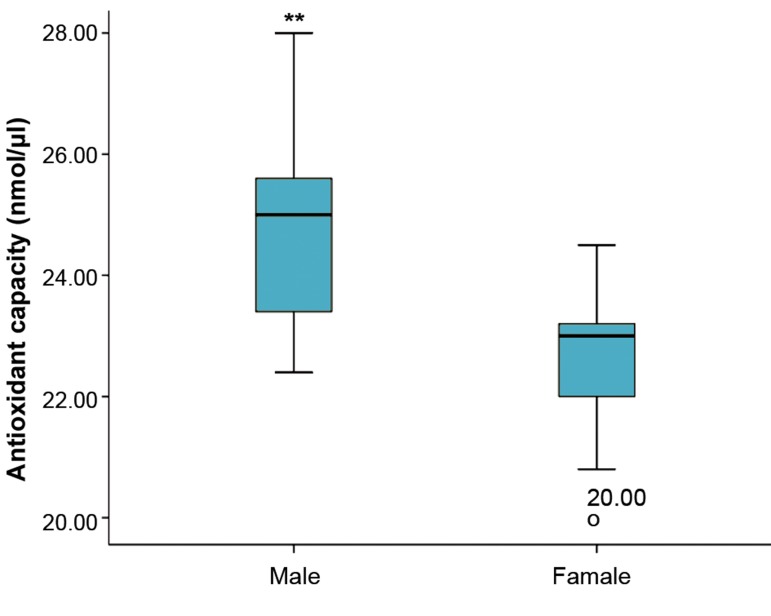
Total antioxidant capacity (TAC) concentration in individual culture media droplets after 48 hours culture of human post compaction embryos. **; Significant difference from female embryos (P<0.001).

Table 1 shows the demographic characteristics of participants. We observed significantly higher glucose consumption by female embryos during the 48 hours embryo culture compared to the male embryos. The mean of glucose concentration consumed by the female embryos (30.7 ± 4.7 pmol/embryo/hour) was significantly higher than that of the male embryos (25.3 ± 3.3 pmol/ embryo/hour, P<0.001, Fig .1).

**Table 1:** Demographic and clinical characteristics of patients


Included women characteristic	Male embryos	Female embryos

The relevant patient number	27	24
Women age mean (Y) (range)	33.7 ± 1.1(23-38)	34.1 ± 0.9(24-38)
FSH levels at baseline (IU/l)	7.2 ± 1.8	7.5 ± 2.1
Mean anti-mullerian hormone levels at baseline (μg/l)	1.8 ± 1.4	1.9 ± 1.2
Total assessed embryos in each group	30	30


Data rare presented as mean ± SE or n. FSH; Follicle stimulating hormone.

**Fig.2:**
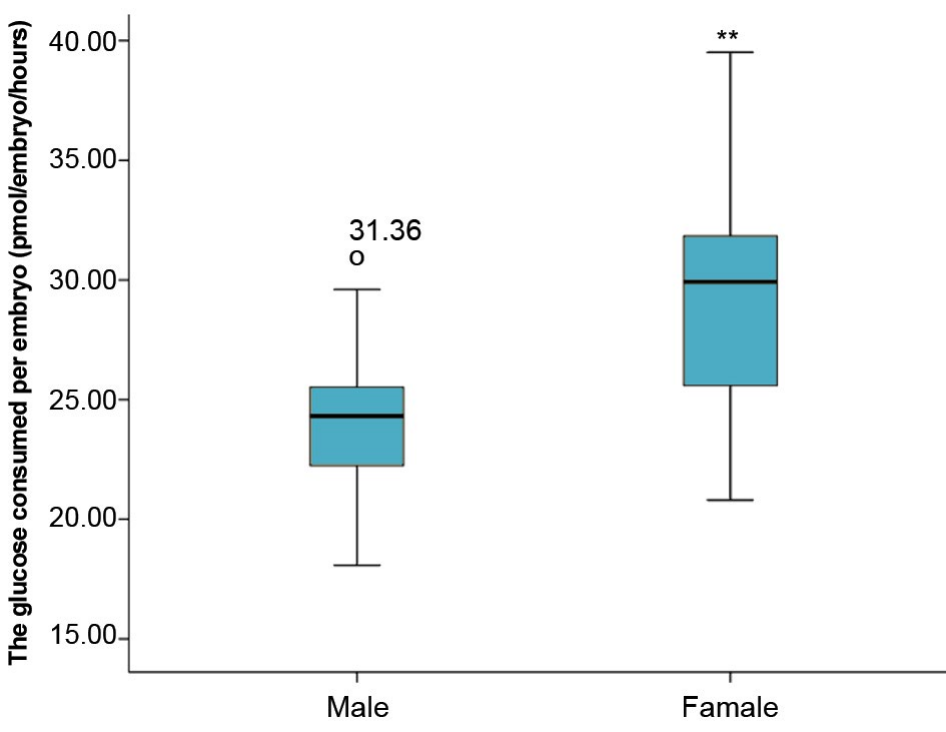
The median glucose concentration consumed by embryo per hour (pmol/embryo/hour). **; Significant difference from female embryos (P<0.001).

Assessment of TAC concentration in the individual embryo culture medium in terms of sex and glucose consumption showed an indirect association between glucose utilization and TAC concentration. There were significantly lower levels of TAC in the surrounding culture medium of female embryos (22.60 ± 0.19 nmol/ μl) compared with male embryos (24.74 ± 0.27 nmol/μl, P<0.01, Fig .2).

## Discussion

Our results indicated significantly more glucose consumption by female embryos on days 4 and 5 of preimplantation development compared to male embryos. This increased consumption in female embryos was concomitant with significantly lower TAC concentration in their surrounding culture medium compared with male embryos.

Animal (4) and human (16) studies previously reported the sex related pattern of glucose consumption in preimplantation embryos. On the other hand, other studies reported varying developmental kinetic rates between male and female embryos. Female embryos experienced slower development rates, which was probably due to increased glucose uptake (17).

It seems that differential expression of X-chromosome linked genes involved in glucose metabolism, such as SOX, MnSOD, BAX and most importantly G6PD which suggest to be stress inducing factor and is significantly higher in female embryos than males, is the cause for the observed difference in developmental kinetic rate between male and female (18). Researchers proposed that OS induction followed by generation of ROS was the probable mediator between slow development rate and high glucose metabolism (19). Impaired glucose metabolism as seen in diabetes could lead to decreased superoxide dismutase (SOD) and glutathione S transferase (GST) expression as important antioxidant enzymes (20). Induced OS might alter the cell signaling pattern and metabolism (21). OS could affect the genome and epigenome in the form of DNA, RNA, proteins and microRNAs. The slow developmental kinetic rate of female embryos in the presence of OS was not the result of mitosis reduction. Rather, the impaired proportion of blastocyst inner cell mass (ICM) reduction in favor of trophectoderm (TE) enhancement could be a possible cause (22).

ROS generation, which is one main feature of aerobic metabolism and mitochondrial oxidative phosphorylation, originates from various sources both inside the embryo as well as the embryo’s surrounding medium. In the embryo, reductions in mitochondrial oxygen generate ROS via multiple enzymatic mechanisms during normal metabolism; this increasing concentration of ROS can activate the antioxidant defense mechanism (21).

Cleaving embryos before compaction utilize lactate and pyrovate during glycolysis as an anaerobic metabolism, therefore the production of ROS could be minimize, whereas glucose consumption around the time of compaction employed the oxidative phosphorylation which could lead to increased production of ROS due to aerobic metabolism of glucose (5). Under such circumstances the antioxidant defense system would protect cells from damage until the over production of ROS overcome the antioxidant defense.

The counteractive antioxidant system is linked both to extra and intra embryonic circumstances. Extra embryonic conditions present as non-enzymatic antioxidants in follicular and tubal fluids, as well as the embryo culture medium. Intra embryonic protection is mainly comprised of enzymatic antioxidants (22). In the IVC systems as with in vivo media, the redox potential of antioxidant compounds that have ROS trapping ability is very important. A major antioxidant compound of embryo culture media is EDTA, a metal chelator, which is supposed to inhibit both enzymatic and non-enzymatic oxidation. Another known antioxidant compound in culture media, albumin, contains proxyabsorbing potency that can trap ROS. However, culture media are closed systems unlike dynamic systems such as tubal and follicular fluids present in the female genital tract which can exchange antioxidant compounds with cells (23). In this environment, progressive production of ROS results from increasing oxidative phosphorylation; glucose metabolism may induce OS which can lead to subsequent damage. Antioxidants inhibit oxidation of macromolecules via ROS removal; in this way they are subjected to oxidation (24) and concentration decline.

Our data showed significantly less TAC concentration in the culture media at 48 h post-compaction of the female embryo culture along with increased glucose utilization compared to male embryos. We could not measure the ROS content of the culture medium droplet because of the inadequate sample size (20 μl) which was not sufficient for simultaneous determination of glucose, TAC and ROS. However, previous reports of increased ROS production attributed to further glucose metabolism indicated that the higher amount of ROS seen in the female embryo culture medium was not out of context. Therefore, we analyzed the antioxidant status of the remaining medium that surrounded the embryo in order to assess OS, for the first time, with regards to glucose uptake and embryo sexuality.

In this study, for the first time the glucose consumption by individual embryos was evaluated indirectly and noninvasively through measuring the remnant glucose in culture medium after embryo incubation period. In this way, the embryos remain intact; therefore this technique can be used for similar purposes in the clinic. However, due to the limited, closed condition of culture systems, OS induction is unavoidable. On the other hand, addition of antioxidant compounds must be logical and based on accurate observations because of the toxicity of excess chemical compounds and antioxidants. However, we have evaluated a relatively small number of embryos because of the limitations in patient inclusion criteria and the use of only one ART center for sample collection.

## Conclusion

The results of this study could be of benefit in two areas-first, these results might improve knowledge of sex related metabolic differences and modification of embryo culture mediums based on embryo requirements. Second, such information following other related observations could be used for non-invasive recognition of embryo gender before transfer in IVF/ICSI cycles.

In this study, we did not predict the embryo sexuality before PGD, but we presented the potential variable that its value was significantly associated with embryo sex determined previously by PGD. We suggest that considering such metabolic variables can help us in noninvasive prediction of pre-implantation human embryo sex. However to confirm our findings as well as other observations from similar studies, it would be necessary to design detailed studies with higher numbers of samples.
